# Patient Perspectives on Health Data Privacy and Management: “Where Is My Data and Whose Is It?”

**DOI:** 10.1155/2018/3838747

**Published:** 2018-12-02

**Authors:** Mart Wetzels, Eva Broers, Peter Peters, Loe Feijs, Jos Widdershoven, Mirela Habibovic

**Affiliations:** ^1^Department of Industrial Design, Eindhoven University of Technology, Eindhoven, Netherlands; ^2^Department of Medical and Clinical Psychology, Tilburg University, Tilburg, Netherlands; ^3^Department of Medical and Clinical Psychology, Department of Cardiology, Tilburg University & Elisabeth TweeSteden Hospital, Tilburg, Netherlands

## Abstract

New technologies are increasingly evaluated for use within the clinical practice to monitor patients' medical and lifestyle data. This development could contribute to a more personalized approach to patient care and potentially improve health outcomes. To date, patient perspective on this development has mostly been neglected in the literature. Hence, this study aims to shed more light on the patient perspective on health data privacy and management. Focus groups with cardiac patients were done at the Elizabeth TweeSteden Ziekenhuis (ETZ) in the Netherlands as part of the DoCHANGE project. The focus groups were conducted using a semistructured protocol which was organized around three themes: privacy regulations, data storage, and transparency and privacy management. Five focus groups with a total of 23 patients were conducted. The majority of the patients preferred to have access to their medical data; however, the knowledge on who has access to data was limited. Patients indicated that they do not want to share their medical data with health insurance companies or the pharmaceutical industry. Furthermore, most patients do not see the added value of supplementing their medical dossier with lifestyle data. Current findings showed patients prefer access to and control over own data but that the knowledge concerning data privacy and management is limited. Sharing of non-medical health data (e.g.,, physical activity) was considered unnecessary. Future studies should address patient preferences and develop infrastructure which facilitates medical data access for patients.

## 1. Introduction

Currently, advanced technologies are available for patient self-monitoring including both medical (e.g., cardiac functioning) [1] and lifestyle parameters (e.g., physical activity [2], sleep [3]) [4]. These devices can collect significantly more data than is needed for disease management only [5]. Consequently, a huge amount of health data is coming in on a daily basis. This is considered a positive development, as these data can give more detailed insight into patients' functioning and perhaps provide a possibility of tailoring the care more to patients' needs [6]. However, important concerns, from different perspectives, are being raised regarding the storage, privacy, visibility, and usage of these data [7].

The confidentiality to the patient-physician relationship is essential in the patient perspective on health data sharing [8]. Kane et al. described that patients want to have full access to their medical data and have control over who has access to it. One-third of patients in primary care want to be informed if their medical information is shared among health care professionals. Others want the medical data to be instantly available to their health care providers [8]. Patients indicate to be more willing to share anonymised and insensitive data (e.g., limited information about their current health problem) than full current and past medical/health information including potentially sensitive problems (e.g., mental health) [8]. Furthermore, the willingness to share health data is greatly influenced by the nature of the recipient, with patients unwilling to share data with researchers, administrators, or other governmental institutions, but generally willing to share with health care professionals [8]. Whether this goes for all health data (medical versus lifestyle) and all health care professionals remains unknown and leads to other important questions that are still unanswered: who should be managing which health data, who should be able to access them, and under what conditions?

Hence, the current study was designed to examine (1) whether patients are aware of where their health data is stored and who can access it, (2) what patients' preferences are regarding medical data storage, privacy, and management, and (3) what patients' preferences are regarding collection and sharing of lifestyle data.

## 2. Methods

### 2.1. Participants

Patients diagnosed with coronary artery disease (CAD), heart failure (HF), or hypertension (HT) were approached for participation. Also, patients had to have at least two of the following risk factors: smoking, positive family history, increased cholesterol, diabetes, sedentary lifestyle, and psychosocial risk factors. Further inclusion criteria were as follows: age 18-75 years, access to the Internet (and sufficient knowledge on using a personal computer or smartphone), and adequate knowledge of the Dutch language. Additional inclusion criteria for HF patients only were as follows: left ejection fraction of ≤ 35% and experience HF symptoms (e.g., shortness of breath, chest pain, and exhaustion). Exclusion criteria were defined as significant cognitive impairments (e.g., dementia), being on the waiting list for heart transplantation, life expectancy less than 1 year, life-threatening comorbidities (e.g., cancers), and a history of psychiatric illness other than anxiety/depression.

### 2.2. Procedure

Patients were approached for participation by their cardiologist during outpatient visits. All eligible patients were provided with information about the study both orally and in writing. Within a week after receiving the information, patients were contacted by telephone and enrolled in the study (if they wanted to). Patients received a letter indicating the date, time and the location of the focus group meeting. Upon arrival at the ETZ, patients were requested to sign the informed consent before starting the focus group. After the meeting patients were offered parking costs refund and a 10 euro worth gift card. The study was approved by the Medical Ethics Board of the ETZ.

### 2.3. Study Outline

The study described a substudy of the ongoing Do Cardiac Health Advanced New Generation Ecosystem (DoCHANGE) clinical trial which is registered at https://www.clinicaltrials.gov (NCT02946281) [9]. The DoCHANGE study aims to support cardiac patients (as defined in the inclusion criteria below) in lifestyle change and disease management by providing them with new technological solutions combined with behavior change techniques. One of the aims of the DoCHANGE trial is developing a system to empower patients concerning their medical/lifestyle data management and develop a personal data storage. Hence, current focus groups were performed to examine patients' views and preferences.

Five 90 minutes (2 x 45 minutes) focus groups [10] were conducted at the ETZ and were facilitated by two moderators (MH, MW, or EB). Groups were designed to include no more than eight patients during each meeting. Including more patients might discourage self-disclosure of some patients [11]. The study was conducted over a period of 4 months. The sample size that was needed to reach data saturation was not known a priori. For qualitative data, there are no exact sample size requirements, as indicated by the World Health Organization guidelines this mainly depends on the saturation of information [12].

The moderators used a semistructured interview guide with questions. Before starting the meeting, patients were asked to fill in a brief questionnaire—addressing their demographic variables (age, gender, education, marital status, and working status)—and to answer three questions concerning medical data storage, privacy, and management. These questions were used to direct the patients towards the subject of the focus group and to stimulate self-disclosure regarding this topic. After this, the participants were asked to briefly introduce themselves and share their cardiac history with the group. The questions that followed aimed to get insight in (1) patients current knowledge regarding medical data storage, privacy and management, (2) patients preferences regarding medical data storage, privacy and management, and (3) patients preferences regarding collection and sharing of lifestyle data.

Each focus group was audio recorded and transcribed. The focus group sessions took place in a meeting room of the hospital at the outer wing of the hospital that is primarily used for conferences and meetings.

### 2.4. Data analysis

The transcripts of each focus group were analyzed by three independent readers (MH, MW, and EB). Open coding was used to isolate themes according to guidelines of thematic analysis [13]. The transcripts were analyzed by hand, and the analysts met to discuss the themes that independently emerged. Final themes were only included if approved by all the analysts.

## 3. Results

The focus groups consisted of 5 sessions with a total number of 23 patients included. The majority of patients were male (N=16). The mean age of the population was 67,2 years. The results section is structured based on the questions in the protocol and quotes from patients are highlighted where appropriate. The term “data” refers to both medical data and personal (wellbeing/lifestyle) data which were not specified otherwise.

*How Is Data Protected When Stored?* In general, patients believe their medical data is protected by the hospital, or other care providers, who generate the data.* “You do assume that your doctor handles anything you share with care. It's a relationship built on trust. If such as system is hacked, there is not much you can do about it.” (M58)* Also, this information is shared among other health practitioners upon their request. Some patients stated that they think the mechanism to share medical data is limited. One patient raised the concern of their medical data being sold:* “The general practitioners have a system in the Netherlands that is owned by an American Holding. They are the eventual owner of the data, and they bundle it up and sell it; hundreds of people at a time.”(M72)* Patients agree that the protection of their data is essential but do not express existing detailed insight on* how* their data is protected at the moment.

*Who Has Access to Your Data?* The majority of participants think their general practitioner (GP) and a specialist, e.g., their cardiologist, have access to their medical file. Additional health practitioners can get access if someone signs it and a secrecy vow warrants the legitimacy. Several patients stated: “*I think the insurance companies also have a lot of information on the invoices they get.”(M72)* The majority of patients did not want the insurance to have access;* “It is very unclear how the commercial side of health care remains separated from the actual care with these systems”(M74)*. The difference between having access to the medical files, blood test, etc., and byproducts, bills of treatments, is highlighted here. Access control is provided for medical files, but it is unclear what information is available, and used, as byproducts of other services like insurance claims.* “I go to a doctor, and he writes the bill that goes straight to the insurances so that they will have this information anyway.”(M58)*

*Is the Security of Your Data up to Your Wishes?* According to most patients, a sense of trust - in the existing system and care providers - is felt to be of crucial importance in these digital systems. No system can guarantee the safety of their files, but they are an inevitability for future care.* “They are probably trying their best, but sometimes things go wrong.”(M76)* Some participants are afraid that without joining the digital health care they might not get care in 10 years.* “I think there will be a moment when you have to. Otherwise, you won't get any health care.”(M58) “I think there is a point where you can no longer fight it. The bigger it becomes, the less of a choice you have but to join.”(F67)* One participant mentions that we are already being tracked and Google already has a lot of data on you whether you like it or not. No patient explicitly stated any real practical wishes.

*Where Is Medical Data Stored?* In general, patients think their medical data is stored at the health care institutions where it is generated.* “I think it is stored in the database of the doctor.” “I assume that the hospital has a big database of all the files. If I'm in an appointment with my doctor, I always see him typing, so that must be going somewhere.”(M58)* Current practices include sharing the medical data between the GP and hospital.* “I think that information that is important to the GP will be transferred to the GP.”(M68)* All patients have been asked to sign agreement forms that other health practitioners can get access to their dossier but some express concern about the transparency of access;* “Who is authorized to see the data? I should know who can and is allowed to view this data.”(M58)*. One patient discussed the process of switching GPs and sharing data:* “When you switch GPs, you ask if you can take your file. They used to make a fuss about that, having it send by mail, etc. Now they just put it in an envelope and give it to you. You can open it and look at it. I think that's your right because they are your files and you can check if they are correct”(M76)*. The latter remark confirms the observation that patients are fine with their medical data being stored at the health care institutions, but would like to be able to have access themselves; for verification and sharing themselves.

*Where Do You Think Medical Data Should Be Stored? *Most patients have two different points of view in this. Data should be stored in trusted locations (like a GP or hospital) and should be strongly linked with other databases.* “There should be central storage for all data where people can take information from”(M76)*. On the other hand, patients argue that there should be only one trusted location where other health practitioners can add information too or request partial access.* “Why would a specialist need access to my entire life? Every specialist has his field and needs his information to do his job. I think that would be best suited for a GP since you have more regular contact with your GP”(F69)*. Some participants propose to have their medical file stored on a card or chip, so they can grant physical access when they want to;* “If we start with a centralized patient system for all medical conclusions. That could be on a chip, and I can take it to my doctor or GP whenever I have a medical issue”(F58)*. The mechanism to have data stored on an object that the patient owns, or has access to, resonates with the results of the previous question.

*What Are Your Preferences; Data Storage in a Central Place in the Hospital or Data under Your Administration Where You Can Decide Who Has Access? *In general, patients want to have their data stored in a central place with the ability to access it.* “On one hand, I like the openness. A specialist also needs a second opinion sometimes, so you should be able to look at the files with multiple people. On the other hand, if patients can make a clear decision about his disease and treatment, he or she should be allowed to decide who has access to the files.”(M48)* The management of access and data appears to be a too large responsibility for patients to handle themselves; they would rather have specialists taking care of that.* “I'm not interested in managing that myself anymore. I'm of certain age and can't be bothered.”(M67) “I think it wouldn't solve anything if you manage it yourself. One person is less precise than the other. If you save it on a computer yourself, how much danger is there someone would break in? Central storage would be better; I would let someone else manage it.”(M64)* The majority of patients would like to have something on them that can provide access to their medical data in case of emergency; ranging from an access mechanism to actual (essential) files.* “If it is an emergency situation, you should be able to access it (the medical files) as soon as possible.”(M48)*

*Do You Have Access To Your Medical Data? *Most patients do not have any, or minimal, access to their medical data.* “I have to rely on what the GP or doctor has on their computer.”(M69)* Some patients indicate that they do not understand why certain information from one health practitioner is not available for another or only limited information is visible to them.* “You question why some things are not in there and if you want to know more there is no more information.”(M68)* Not every participant wants to have access to their medical data although most seem curious to what is written in the files.

*Whom Would You Want to Give Access to Your Medical Data? *In general, patients feel that every health practitioner that is involved in their care (GP, nurses, etc.) should have access to files.* “If doctor X refers me to the psychologist, he is allowed to know what was discussed; I think that is important.”(F56)* Many patients state that “the hospital” can have access to their data without specifying which people of the hospital; or everyone.* “We talked about hospitals being able to view all data but who is the hospital? I think the acting or advising specialist/doctor should have access to the data.”(M66)* Several patients argued for added effort of managing access to their medical data:* “I do wish hospitals would have access to this information. They do make some fuss about privacy which makes me go through an examination twice. This information should be available to them without a problem.”(M66) “My background and medical history are important, it saved me a lot of trouble, so I'm less concerned with protection. I do think that parties who have a financial interest should never get access.”(M74)*

*Whom Would You NOT Give Access to Your Medical Data? *Most patients agree that the health insurances should not have access to their medical files.* “I want to help and think if we can do things cheaper if my health care can be cheaper, but I don't want the insurance companies to have a say about the quality or measure of treatment.”(F68)* Parties with financial gain, from commercial businesses, should not have access to medical data. However, participants think they already have access without them knowing explicitly. The access to pharmacies differ with participants, and some feel that the pharmacist is a service to dispense medication, whereas others feel that they need limited access to provide advice for their medication about their disease.* “Pharmacies are purely about medication; they have nothing to do with the rest of your medical file. The pharmacy should know what medication is combined, so that part of the data is important to them.”(M58)* As mentioned in other questions, granular access control would provide a sense of control over who can access their medical data.

*Would You Want to Have the Control to Grant People Access to Your Medical Data? *Most participants agree that access should be granted on a need to know basis and they would like to be able to manage access. However, the need for access can also be interpreted as a need to have insights on who is accessing their medical files.* “I don't need to manage it but do want to know who and when they check my file. That way I can decide whether grant access or not.”(M64)* An elaborate protocol should be in place to grant access rights when an individual is not able to grant access.* “There comes the point when it's not a possibility (to manage access), and you need someone else to do it. If you have kids or a partner willing to do that it's great, but that's not always the case.”(M62)* In most cases, participants would like to grant family members, or their GP, full access control in case of emergencies. Some specific professionals should always be able to access the medical files, like the GP and treating physician.* “The doctor should be able to access it at all times, in case of emergency. If a hospital needs the files, they should have access.”(F56)* Some participants feel that the responsibility of access control is too large for an individual to manage and that this should be delegated to the GP or another trustee.

*Do You Use any Lifestyle Devices or Other Tools? *Some patients claim not to use any devices and do not see the value of doing so.* “I have a step counter on my phone but I hardly ever use it. Now and then it congratulates me when I took a couple of thousand steps.”(M48)* Those participants would use devices if recommended by the cardiologist but would only use them for a limited amount of time.* “If he would advise it. It's like the cardiologist worked hard to get me healthy again. I want to maintain that.”(M69)* Other participants shared that they use a blood pressure monitor and scale on a regular basis. Participants with diabetes check their glucose levels on a regular basis. The consensus is that people do not use other devices, except an occasional weight check-up and blood pressure measurement.* “If I opened my eyes in the morning and had a good night's sleep, I don't need any machines diagnosing my apnea. If it helps my wife to worry less, I might do it. I think it is a terrible idea to have a machine beneath my mattress.”(M76)*

*Would You Want Non-Medical Data, from Your Lifestyle Devices, to Be Available to Your Cardiologist? *Participants do not see the value of adding all non-medical data to their medical files.* “It's your responsibility. It gives you some personal insight. That doesn't have to be shared with doctors etc.”(M76)* Doing so would burden the cardiologist with more data to analyze.* “They are busy enough. This would just add to that”(F62)* It is unclear who would be responsible for monitoring this data.* “A GP doesn't have time to check all that, unless there is an alarm system that triggers with divergent or alarming results.”(M68)* Another argument provided against sharing data with the cardiologist is the fear of being reprimanded; especially with food logs. Some participants would like to have all data available to the cardiologist to have it available when needed.

*Who Could Get Access to Your Non-Medical, Lifestyle, Data and Why? *It is not a common idea that lifestyle data is even shared, let alone stored in a medical file.* “If a specialist needs this information with a certain purpose, then I would share it. But why would he need it?”(M69)* However, the GP seems to be the central medical person to have access to this data.* “I have medication for my blood pressure, and I like that the GP has that on file. I check my blood pressure but don't share that.”(M62)* In general, participants question the need for medical personnel to have access to that type of data.* “I think it's a step too far if data about my activity and fluid intake go into my medical file.”(M58)*

*Who Could NOT Get Access to Your Non-Medical, Lifestyle, Data and Why? *Participants do not see why data would be stored.* “I think this gets very close to your privacy. I do the best I can, taking my personal life into account. The data regarding my exercise etc. are very personal in that sense. I' m not waiting for a specialist or GP to judge me in regards to that data.”(F58)* They can manually record and keep track of their performance when doing fitness or record their fluid intake on paper. A digital record is not needed, some even call it childish. As with medical data, participants are afraid that the insurance company will be able to access this data and that this will influence their reimbursements or premium.* “It does bring a risk if all this information is also stored. Your cardiologist, but also the insurance companies could get information about your activity level.”(M69)*

## 4. Discussion

The current study has tapped into a critical topic of health data privacy and management, namely, the patient perspective. The results indicate that patients are not sufficiently informed about where their data is stored and who has access to their data. Furthermore, the majority of the patients reported that they would want to have access to their data and that they are reluctant to share their data with for example insurance companies; similar to the results of a questionnaire study [14]. Concerning lifestyle data patients indicated that they do not see the added value of sharing these data with their health care providers. Although they might be interested themselves to have these data, sharing them with a health care provider is perceived as unnecessary. Current results also showed that patients would prefer to have control over their data and to decide who should be granted access and when.

Limitations must be acknowledged. No quantitative data were obtained. Hence, it was not possible to indicate which percentage of patients had what perspective. However, this study tapped into the patient perspective concerning data privacy and management which is an understudied and neglected perspective.

The focus groups on the topic of privacy concerning medical data are needed in the ever-evolving digital age where new regulations such as the European General Data Protection Directive (GDPR) [15] are instated to protect the data of individuals. New technologies enable monitoring and aggregation of detailed personal information. The transparency on the use of the personal data of these systems, however, is mostly lacking. Based on the results presented in this paper, it is evident that patients are not as informed about the use and storage of their medical data as they would like. The preference for managing your medical data varies between subjects. The authors hypothesize that patients desire access to their medical data to share with other health practitioners - when the digital system prevents them from doing so - instead of fully managing their data. The problem of the disconnection between the patients and their data can be solved with technology integration. Either existing systems need to be connected, or a new system needs to be introduced. From a societal point of view, it is questionable to make patients responsible for managing data that is (often) not fully understood. Medical information can, to some extent, be documented in a more familiar language to make it more understandable, but nuance can get lost in translation, and such documentation transcends the health practitioners' note-taking efforts.

While prevention of cardiovascular disease and promotion of self-management seem crucial [16] to decrease the incidence, disease burden, and associated health care costs, patients appear to treat lifestyle data differently than medical data. As shown in the current study, patients are willing to share their medical data with their health care providers, but the majority indicated not to see any added value in sharing lifestyle data. Despite the evidence showing that behavioral and lifestyle factors are major predictors of poor health outcomes [17], patients seem not to be sufficiently aware of this importance. Lifestyle factors are still regarded as something “personal” or “private” that does not concern the health care providers. These findings may explain the lack of self-management or responsibility for one's health by the majority of patients [18].

## 5. Recommendations

The relevance of lifestyle factors in health promotion and associated data should be more emphasized. The recommendation would be to work towards a model where lifestyle data is perceived as necessary as all other medical data. Also, models that focus on enhancement of behavior change should be studied to assist the patient in making appropriate changes.

According to the results, patients are not well-informed on privacy and their medical data. Better education and more transparency are required to improve the knowledge of patients. Patients show a high trust in their regular physicians but how confident will patients be with entrusting their data to unfamiliar data officers?

The level of insight, from patients, needs to shift from data being stored “somewhere in the cloud” to “at the data storage of the health practitioner who created the data”. The patients can be empowered by involving them in decision making concerning data and privacy but not by making them responsible.

Future work should evaluate new designs and implementations of data management systems [19] that address privacy for medical data instead of obtaining more information from a larger population on the same topic.

## Data Availability

Audio recordings are not publicly available but can be delivered upon request.
